# HIV testing during pregnancy for prevention of mother-to-child transmission of HIV in Ethiopia

**DOI:** 10.1371/journal.pone.0201886

**Published:** 2018-08-09

**Authors:** Yohannes Ejigu, Biniyam Tadesse

**Affiliations:** 1 Department of Health Economics, Management and Policy, College of Health Sciences, Jimma University, Jimma, Ethiopia; 2 International Center for Health Monitoring and Evaluation (ICHME), Jimma University, Jimma, Ethiopia; University of Liverpool Institute of Infection and Global Health, UNITED KINGDOM

## Abstract

**Introduction:**

HIV testing during pregnancy provides an entry point to prevention of mother-to-child transmission of HIV and to access treatment for HIV positive women. The study aimed to assess the uptake of HIV testing during pregnancy and associated factors among Ethiopian women.

**Methods:**

We analyzed the 2016 Ethiopian Demographic and Health Survey dataset. Women who gave birth within one year prior to the survey were included in the analysis. Uptake of HIV testing during pregnancy is defined as receiving HIV testing service during pregnancy and/or at the time of delivery and knew the test results. Adjusted odds ratios (AORs) and 95% confidence intervals (95% CIs) were calculated by using step-wise backward logistic regression analyses to identify factors associated with HIV testing during pregnancy.

**Results:**

A total of 2114 women who were pregnant in the last one year prior to the survey were included in the analysis. Of these, only 35.1% were tested for HIV and received the test results during pregnancy. About one third of women who had antenatal care follow-up missed the opportunity to be tested for HIV. Compared to women who had no formal education, those who had primary level education (AOR = 1.55; 95% CI: 1.12–2.15), secondary level education (AOR = 2.56 95%CI: 1.36–3.82), or higher education (AOR = 3.95, 95%CI: 1.31–11.95) were more likely to be tested for HIV during pregnancy. Similarly, having awareness about mother-to-child transmission of HIV (AOR = 2.03, 95%CI: 1.48–2.78), and living in urban areas (AOR = 3.30, 95%CI: 1.39–7.85) were positively and independently associated with uptake of HIV during pregnancy. Women who have stigmatizing attitude towards HIV positive people were less likely to be tested for HIV (AOR = 0.57, 95%CI: 0.40–0.79).

**Conclusion:**

Uptake of HIV testing during pregnancy is low. Missed opportunity among women who had antenatal care visits was very high. Integrating HIV testing with antenatal care services, improving HIV testing service quality and access are essential to increase uptake of HIV testing during pregnancy and reach the goal of eliminating MTCT.

## Introduction

Globally an estimated 1.8 million children infected with HIV in 2016 [1], and over 90% of infections among infants and children occur through mother-to-child transmission (MTCT) during pregnancy, labor/delivery, or breastfeeding [2]. The effectiveness of zidovudine in prevention of mother-to-child transmission of HIV (PMTCT) has been recognized in the early 1990^th^ [3], and combination of antiretroviral drugs further reduces vertical transmission of HIV [4, 5]. As a result, notable progress has been made in PMTCT in both developed and developing countries [6].

HIV testing during pregnancy provides an entry point to PMTCT, treatment, care and support services when women are diagnosed with HIV [7]. However, low uptake of HIV testing has been a bottleneck for PMTCT and subsequent HIV treatment, care and support services. An estimated 30% of livings with HIV were unaware of their HIV status at the end of 2016 [8]. Previous studies have identified different barriers to uptake of HIV testing, which includes poor knowledge about MTCT, low maternal education level, fear stigma, and poor access to health services [9–11].

Ethiopia is one of the sub-Saharan African countries with high burden of HIV; an estimated 71,000 people live with HIV in 2016 [6]. Ethiopia started universal HIV screening of pregnant women in 2007 [12], and currently, it is committed to eliminate MTCT of HIV by 2020 and achieve the 90-90-90 treatment targets endorsed in the 2016 United Nations political declaration on ending AIDS [13, 14]. However, coverage of HIV testing during pregnancy should be at least 95% to achieve the goals [15]. In Ethiopia, almost all HIV testing during pregnancy have been provided as part of antenatal care services and only two third of pregnant women had antenatal care visits [16], which can be a bottleneck to increase uptake of HIV testing during pregnancy.

Previous Ethiopian studies assessing uptake of HIV testing during pregnancy identified socio-demographic and health system related factors influencing HIV testing during pregnancy [17–20]. However, these studies were conducted among women visiting health facilities and pregnant women who did not have access to health facilities were left out. Moreover, the studies cover limited geographic scope. As to our knowledge, there was no population based national study addressing this major public health issue in Ethiopia. Therefore, the aim of our study was to assess uptake of HIV testing during pregnancy and associated factors in Ethiopia using nationally representative data from Ethiopian Demographic and Health Survey 2016 (EDHS).

## Materials and methods

We used data from the EDHS 2016 survey. The survey was conducted between January 18, 2016 and June 27, 2016. It covered all nine regions and two city administrations of Ethiopia. The survey was designed to be representative of the country and each region by taking a sampling frame from the 2007 population census of Ethiopia. The current study focuses on analyzing coverage of HIV counseling and testing service during pregnancy with a primary purpose of preventing mother-to-child transmission of HIV.

### Sampling strategy and data collection field work

The survey employed a stratified, two-stage cluster sampling technique. Ethiopia has nine administrative regions and two city administrations. The regions are divided in to zones, districts and *kebeles* (the lowest administrative units). A total of 84,915 census enumeration areas (EAs) were created by dividing *kebeles* with an average of 181 households per EA. Samples of 645 EAs (202 from urban and 443 from rural areas) were randomly selected, and 28 households from each EA were included in the survey by applying a systematic random sampling strategy. The survey administered questionnaires and collected biological sample from women of reproductive age group (15 to 49 years), children under five years old and men age between 15 to 59 years.

However, for this particular study, women of reproductive age group were the source population, and women who gave birth to their last child in the last one year prior to the survey were the study populations (we restricted the sample to birth in the last one year so as to minimize recall bias).We used data collected from women of reproductive age group (15–49 years) using DHS’s women’s questionnaire [21], The questionnaire was a standardized and field tested tool used in different countries and only a subset of variables were included in the current study. Data quality was preserved by pre-testing the survey tools preceding the survey, and data were collected by trained survey teams consisted of one team supervisor, one field editor, four female interviewers, and two male interviewers. In addition, independent quality control team was used to check the quality of the collected data.

### Outcome variable

The outcome variable is uptake of HIV testing during pregnancy (yes or no). Uptake HIV testing during pregnancy is defined as receiving HIV testing service during pregnancy and/or at the time of delivery and knew the test results.

### Predictor variables

We included potential predictors of HIV counseling and testing, such as maternal age in years, marital status (never married, married and divorced/widowed/separated), religion (Orthodox, Muslim, Protestant and Others), region, residence (urban and rural), educational status (no education, primary, secondary, and higher), wealth index (poorest, poorer, middle, richer, and richest), and awareness of MTCT of HIV was defined as awareness of women about possibility of HIV transmission from HIV positive mother to a child (yes or no). Employment status was defined as having any kind of job other than their housework in the last 12 months before the survey (not employed, or employed). Finally, stigmatizing attitude towards HIV positive people was measured by respondents’ refusal to buy vegetable form known HIV positive vendor (yes or no).

### Data analysis

The survey employed a stratified two stage sampling and there was over sampling of some populations, as a result complex survey data analysis and weighting were employed as recommended by DHS [22], to make the data more representative of the national population and all the findings are based on weighted analysis. Descriptive statistics were computed to summarize socio-demographic characteristics of study participants. We compared the socio demographic characteristics of women who had HIV testing during their last pregnancy with those who were not tested for HIV using weighted chi-square tests. Moreover, we run a weighted bivariate and multivariate logistic regression analysis, reporting odds ratio (OR) and 95% confidence interval (CI). The multivariate logistic regression analysis was conducted by using step-wise backward elimination technique after including all relevant covariates in the model and eliminating one variable at a time. Variables which had P values <0.05 were considered significant. Data analysis was carried out by STATA version 14 (Stata Corp., College Station, TX).

### Ethical issues

The study was approved by the Federal Democratic Republic of Ethiopia Ministry of Science and Technology and the Institutional Review Board of ICF International. Moreover, the respondents gave written consent to participate in the survey. Data collectors were trained for one month on different aspects of the data collection process, including ethical issues.

## Result

### Socio-demographic characteristics of study participants

A total of 2414 women who gave birth in the past one year prior to the survey were included in the analysis. Majority, 73% were in the age group of 20 to 34 years, and most, 96% were married. About 59% of women have no formal education and 44% were Muslims. Most, 80% were living in rural areas and two third of them were not employed. More than one third, 36% were classified under the poorest income category (Table 1). Women who were tested for HIV during pregnancy were more likely to be educated, urban residents, employed and have highest wealth quintile (Table 1).

**Table 1:** Socio-demographic characteristics of study participants.

Characteristics	Total (n = 2414), n(%)	HIV testing during pregnancy		P value
		Not tested, %	Tested, %	
**Age in years**				
15–19	196(8.1)	70.8	29.2	0.21
20–24	587(24.3)	63.2	36.8	
25–29	705(29.2)	63.7	36.3	
30–34	482(20.0)	61.7	38.3	
35 and above	444(18.4)	70.3	29.7	
**Marital status**				
Never married	16(0.7)	36.0	64.0	0.07
Married/live with partner	2311(95.7)	64.7	35.3	
Widowed/Divorced/Separated	87(3.6)	76.9	23.1	
**Religion**				
Muslim	1065(44.1)	76.2	23.8	<0.0001
Orthodox	793(32.9)	45.9	54.1	
Protestant	473(19.6)	68.8	31.2	
Others	83(3.4)	80.7	19.3	
**Educational status**				
No education	1421(58.9)	75.3	24.7	
Primary	775(32.1)	57.8	42.2	
Secondary	162(6.7)	27.1	73.0	<0.0001
Higher	56(2.3)	10.6	89.4	
**Residence**				
Rural	2118(87.8)	71.5	28.5	<0.0001
Urban	296(12.3)	18.0	82.1	
**Region**				
Affar/ Somali	141(5.8)	83.7	16.3	<0.0001
Tigray	168(7.0)	29.6	70.4	
Amhara	442(18.3)	45.0	55.0	
Oromia	1074(44.5)	79.3	20.7	
Benishangul-Gumuz/ Gambela	31(1.3)	64.4	35.6	
SNNPR	482(20.0)	66.3	33.8	
Addis Ababa/Harari/Dire Dawa	76(3.2)	14.2	85.8	
**Employment status**				
Not employed	1496(62.0)	67.5	32.6	0.05
Employed	918(38.0)	60.9	39.2	
**Wealth index**				
Poorest	546(22.6)	86.6	13.4	<0.0001
Poorer	563(23.3)	74.3	25.7	
Middle	483(20.0)	69.3	30.7	
Richer	432(17.9)	55.0	45.0	
Richest	390(16.2)	26.6	73.4	

P value is based on chi-square tests. The frequency and percentages are based on weighted analysis.

### Awareness of women about MTCT, PMTCT and HIV testing

The study showed that women had awareness gap regarding MTCT of HIV. Only 61% women were aware of MTCT during pregnancy, 65% were aware of MTCT during labour/delivery, and 69% were aware of MTCT during breastfeeding. Women who were aware of MTCT during pregnancy, labor/delivery, or breastfeeding were more likely to be screened for HIV during pregnancy (Table 2).

**Table 2:** Awareness of women about MTCT, and their attitude towards HIV positive people.

Variables	Total, (N = 2414), n(%)	HIV testing during pregnancy		
		Not tested, %	Tested, %	p. value
Aware of MTCT during pregnancy				
Yes	1472(61.0)	57.1	42.9	<0.0001
No	942(39.0)	77.3	22.8	
Aware of MTCT during delivery				
Yes	1570(65.0)	57.3	42.7	<0.0001
No	844(35.0)	79.2	20.8	
Aware of MTCT during breastfeeding				
Yes	1671(69.2)	56.8	43.2	<0.0001
No	743(30.8)	83.3	16.7	
Have stigmatizing attitude towards HIV positive people				
Yes	738(30.6)	74.0	26.0	<0.0001
No	1676(69.4)	44.3	55.7	

The table is based on weighted analysis. MTCT: mother to child transmission of HIV

**Fig 1:**
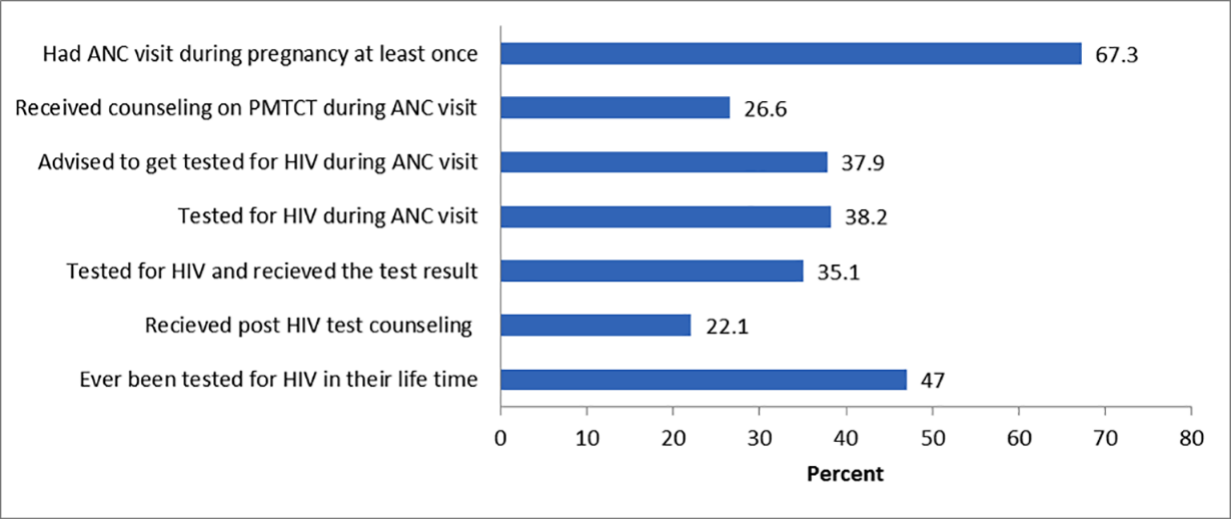
Uptake of ANC, HIV counseling and testing services. ANC: antenatal care. PMTCT: prevention of mother-to-child transmission of HIV. All percentages are calculated from the total number of women. Ever been tested for HIV in their life time include HIV testing during last pregnancy.

The study showed a considerable missed opportunity along the cascade of maternal health care services during pregnancy. As can be seen from Fig 1, only 35% of women were tested for HIV and received the test results although two thirds, 67% of women had at least one ANC visit during their last pregnancy. About one third of women who had antenatal care follow-up missed the opportunity to be tested for HIV. Moreover, only 22% women received post HIV test counseling (Fig 1).

### Factors associated with HIV testing during pregnancy

In bivariate analysis, marital status, religion, level of education, place of residence, region, employment status, wealth, awareness of MTCT during pregnancy and having stigmatizing attitude towards HIV positive people were significantly associated with HIV testing during pregnancy. In multivariate logistic regression analysis, level of education, marital status, place of residence, region, wealth, awareness of MTCT during pregnancy and having stigmatizing attitude towards HIV positive people remained significantly associated. The associations of HIV testing with employment status and religion which were observed in bivariate analysis were attenuated in multivariate analysis.

Having formal education was positively associated with HIV testing during pregnancy. Compared to women who had no formal education, those who had primary level education (AOR = 1.55; 95% CI: 1.12–2.15), secondary level education (AOR = 2.56 95%CI: 1.36–3.82), or higher education (AOR = 3.95, 95%CI: 1.31–11.95) were more likely to be tested for HIV during pregnancy. Moreover, women who were aware of MTCT during pregnancy were twice more likely to be tested for HIV during pregnancy (AOR = 2.03, 95%CI: 1.48–2.78), however, women who had stigmatizing attitude towards HIV positive people were less likely to be tested for HIV (AOR = 0.57, 95%CI: 0.40–0.79). Place of residence and region were also significant predictors of uptake of HIV testing during pregnancy. Women who live in urban areas were three times more likely to be tested for HIV during pregnancy compared to rural residents (AOR = 3.30, 95%CI: 1.39–7.85). Similarly, Compared to women who live in predominantly pastoralist regions (Afar/Somali), those who live in predominantly agrarian regions (Tigray and Amhara), or those who live in predominantly urban regions (Addis Ababa/Harari/Dire-Dawa) were more likely to be tested for HIV during pregnancy. The likelihood of being tested for HIV increase as wealth index increased from the poorest to the richest category. Women in richest category were six times more likely to be tested for HIV as compared to women in the poorest category (AOR = 5.84, 95% CI: 2.99–11.43) (Table 3).

**Table 3:** Factors associated with HIV testing during pregnancy.

Characteristics	HIV testing during pregnancy		Unadjusted OR (95% CI)	Adjusted OR (95% CI)
	Not tested, %	Tested, %		
**Age in years**				
15–19	70.8	29.2	1	—
20–24	63.2	36.8	1.42(0.88–2.27)	
25–29	63.7	36.3	1.39(0.86–2.24)	
30–34	61.7	38.3	1.51(0.89–2.55)	
35 and above	70.3	29.7	1.03(0.61–1.71)	
**Marital status**				
Never married	36.0	64.0	1	1
Married/live with partner	64.7	35.3	0.31(0.64–1.48)	0.23(0.04–1.21)
Widowed/Divorced/Separated	76.9	23.1	**0.17(0.03–0.89)**	**0.07(0.01–0.46)**
**Religion**				
Muslim	76.2	23.8	1	—
Orthodox	45.9	54.1	**3.77(2.52–5.66)**	
Protestant	68.8	31.2	1.45(0.92–2.28)	
Others	80.7	19.3	0.76(0.24–2.46)	
**Educational status**				
No education	75.3	24.7	1	1
Primary	57.8	42.2	**2.22(1.67–2.95)**	**1.55(1.12–2.15)**
Secondary	27.1	73.0	**8.22(4.69–14.39)**	**2.56(1.36–4.82)**
Higher	10.6	89.4	**25.62(8.57–76.57)**	**3.95(1.31–11.95)**
**Residence**				
Rural	71.5	28.5	1	1
Urban	18.0	82.1	**11.47 (6.48–20.32)**	**3.30(1.39–7.85)**
**Region**				
Affar/ Somali	83.7	16.3	1	1
Tigray	29.6	70.4	**12.23 (7.50–19.95)**	**9.55(5.17–17.64)**
Amhara	45.0	55.0	**6.30(3.94–10.06)**	**4.16(2.22–7.79)**
Oromia	79.3	20.7	1.34(0.82–2.22)	0.79(0.43–1.46)
Benishangul-Gumuz/ Gambela	64.4	35.6	**2.85(1.72–4.73)**	1.80(0.92–3.50)
SNNPR	66.3	33.8	**2.62(1.71–4.03)**	1.53(0.79–2.95)
Addis Ababa/Harari/Dire Dawa	14.2	85.8	**31.21 (19.04–51.14)**	**3.55(1.83–6.90)**
**Employment status**				
Not employed	67.5	32.6	1	—
Employed	60.9	39.2	**1.33(1.00–1.79)**	
**Wealth index**				
Poorest	86.6	13.4	1	1
Poorer	74.3	25.7	**2.24(1.42–3.53)**	**2.32(1.47–3.66)**
Middle	69.3	30.7	**2.87(1.75–4.70)**	**3.27(1.94–5.52)**
Richer	55.0	45.0	**5.30(3.29–8.53)**	**5.43(3.31–8.89)**
Richest	26.6	73.4	**17.84(10.39–30.34)**	**5.84(2.99–11.43)**
**Aware of MTCT during pregnancy**				
No	77.3	22.8	1	1
Yes	57.1	42.9	**1.67(1.27–2.18)**	**2.03 (1.48–2.78)**
**Have stigmatizing attitude towards HIV positive people**				
No	44.3	55.7	1	1
Yes	74.0	26.0	**0.28(0.21–0.36)**	**0.57(0.40–0.79)**

OR: odds ratio. MTCT: Mother-to-child transmission of HIV

## Discussion

HIV testing during pregnancy provides an opportunity to PMTCT services and to initiate lifelong antiretroviral treatment for HIV positive women before disease progress to AIDS. Our study showed that only 35% of women were tested for HIV during pregnancy and a large proportion of women missed the opportunity to be tested for HIV although they had at least one ANC visit during pregnancy. The finding is a far from the recommendation WHO and Ethiopian government which states that all pregnant women attending ANC should be offered provider initiated HIV testing and counseling [23, 24]. The study also revealed that more than half of women have never been tested for HIV in their lifetime, which indicates majority of HIV positive women did not have the opportunity to protect their children from vertical transmission of HIV infection and sought antiretroviral treatment for their own health. Women who are not tested for HIV during ANC follow-up may not get a chance to be tested for HIV during postnatal care visit because only 7% of Ethiopian women visited health facilities for postnatal check-up [16].

Ethiopia is committed to the goal of eliminating HIV infection among children by 2020, however, one of the criteria for elimination of MTCT is achieving 95% coverage of HIV testing among pregnant women [15]. Moreover, to achieve the global 90-90-90 HIV treatment target 90% of HIV positive individuals should know their HIV status. But, our finding of only 35% HIV testing during pregnancy indicates elimination of MTCT by 2020 and the 90-90-90 target doubtful. The low HIV testing coverage could be explained by low level of awareness about MTCT during pregnancy; poor level of integration of HIV testing with routine ANC services and poor quality ANC service which can be demonstrated by large number of missed opportunity to be tested among pregnant women who had ANC visits. Moreover, one third of pregnant women did not have ANC visit during pregnancy which could indicate lack of access to maternal health services. Our finding of HIV testing during pregnancy is very low as compared to neighboring African countries. For example, a national survey in Kenya reported that 95% women attended ANC during their last pregnancy, of these, 93% accepted HIV testing and 98% of those tested received the test results [25]. Similarly other studies also reported higher uptake of HIV testing during pregnancy [26, 27].

Our study also showed that proportion of women tested for HIV during pregnancy was uneven across different regions of the country. The coverage ranges from 16% in Afar/Somali regions to 70% in Tigray region. People in Afar and Somali regions are predominantly pastoralists and they move from place to place to place as a result it could be difficult to provide health services. Similar to our findings, previous reports also indicated a very low reproductive, maternal, neonatal and child health service coverage in Afar and Somali regions as compared to other regions of Ethiopia [28]. The study also revealed, a large gap regarding uptake of HIV testing between urban and rural women (82% versus 29%). In Ethiopia, HIV prevalence is higher in urban than rural areas [13], and on the surface, targeting urban residents looks appropriate. However, more than 80% of Ethiopian population resides in rural areas [29], as a result large number of HIV positive women will be left undiagnosed if rural women have no access to HIV testing. Moreover, different studies demonstrated that universal HIV testing of pregnant women is more cost saving and cost effective in different HIV epidemic contexts as compared to targeted HIV testing approaches [30–32].

Education and awareness of women about MTCT during pregnancy were independently and positively associated with HIV testing. Our findings are consistent with previous studies conducted in Ethiopia [17], other African countries [33, 34]. Similarly, uptake of HIV testing during pregnancy was higher among wealthiest categories although HIV testing is provided free of charge in public health facilities. Similar findings were reported by previous studies [35, 36]. Women who had stigmatizing attitude towards HIV positive people were less likely to be tested for HIV. The finding is consistent with other studies [33, 37, 38].

This study should be understood in light of the following strengths and limitations. The study is based on a population data collected from all over the country with nationally representative sample. However, the following limitations should be highlighted. Respondents may not remember past events, which potentially could introduce recall bias, but we restricted our study to women who gave birth during the past one year prior to the survey to minimize potential recall bias. HIV infected pregnant women who knew their statuses before the current pregnancy do not need another HIV testing. But this may not cause significant effect in the overall result, as the expected proportion of HIV infected women in Ethiopia is below 1.5%.

## Conclusion

In conclusion, uptake of HIV testing during pregnancy in Ethiopia is low, substantial number of pregnant women who had ANC visit missed the opportunity to get tested for HIV indicating poor quality services or poor integration of HIV testing with maternal health services. Moreover, uptake of HIV testing was uneven across regions and it was very low in rural areas. Ethiopia could not eliminate pediatric HIV infection unless HIV testing during pregnancy is scaled up. Individual level factors, such as level of education, awareness of MTCT during pregnancy, having stigmatizing attitude towards HIV positive people and economic status were independent predictors of HIV testing during pregnancy. We recommend integration of HIV testing during pregnancy with ANC services and improving quality of ANC services. Moreover, expanding ANC services to rural areas to improve access and coverage; innovative HIV testing approaches should be designed to reach pregnant women from pastoralist regions. Furthermore, community level education about HIV and MTCT for women of reproductive age group could improve uptake of HIV testing during pregnancy.
